# Data on force-dependent structural changes of chromatin fibers measured with magnetic tweezers

**DOI:** 10.1016/j.dib.2014.08.002

**Published:** 2014-08-06

**Authors:** Fan-Tso Chien

**Affiliations:** Institute of Physics, Academia Sinica, 128 Sec. 2, Academia Rd., Nankang, Taipei 11529, Taiwan, Republic of China

## Abstract

The compaction of chromatin fibers regulates the accessibility of embedded DNA, highly associated with transcriptional activities [1]. Single molecule force spectroscopy has revealed the great details of the structural changes of chromatin fibers in the presence of external exerted force [2–7]. However, most of the studies focus on a specific force regime [2,3,8,9]. The data here show force-extension (FE) traces of chromatin fibers as measured with magnetic tweezers, covering the force regime from 0 pN to 27 pN. Those traces provide information for further studies at varied force regimes.

**Table:** 

**Specifications table**	
Subject area	Biophysics
More specific subject area	Chromatin biology, force spectroscopy
Type of data	Graph, figure
How data was acquired	Magnetic tweezers
Data format	Raw
Experimental factors	Chromatin fibers were reconstituted with histone octamers purified from chicken erythrocytes and DNA fragments containing 25 copies 601 nucleosome positioning elements.
Experimental features	Magnetic beads tethered chromatin fibers were stretched with magnetic tweezers at a force range of 0–27 pN
Consent	The data were fully published and allowed to reuse.
Data source location	Institute of Physics, Leiden University, The Netherlands
Data accessibility	The raw data files are provided in the Data in Brief DataVerse, doi:10.7910/DVN/26868[10]

## Value of the data

• The FE data are good candidates for model testing capturing structural changes.
• The number of nucleosomes is certain.
• The data open possibilities to investigate previously unreported mechanical properties of one turn wrapped nucleosome fibers.

## 1 Experimental design, materials and methods

**Fig. 1:**
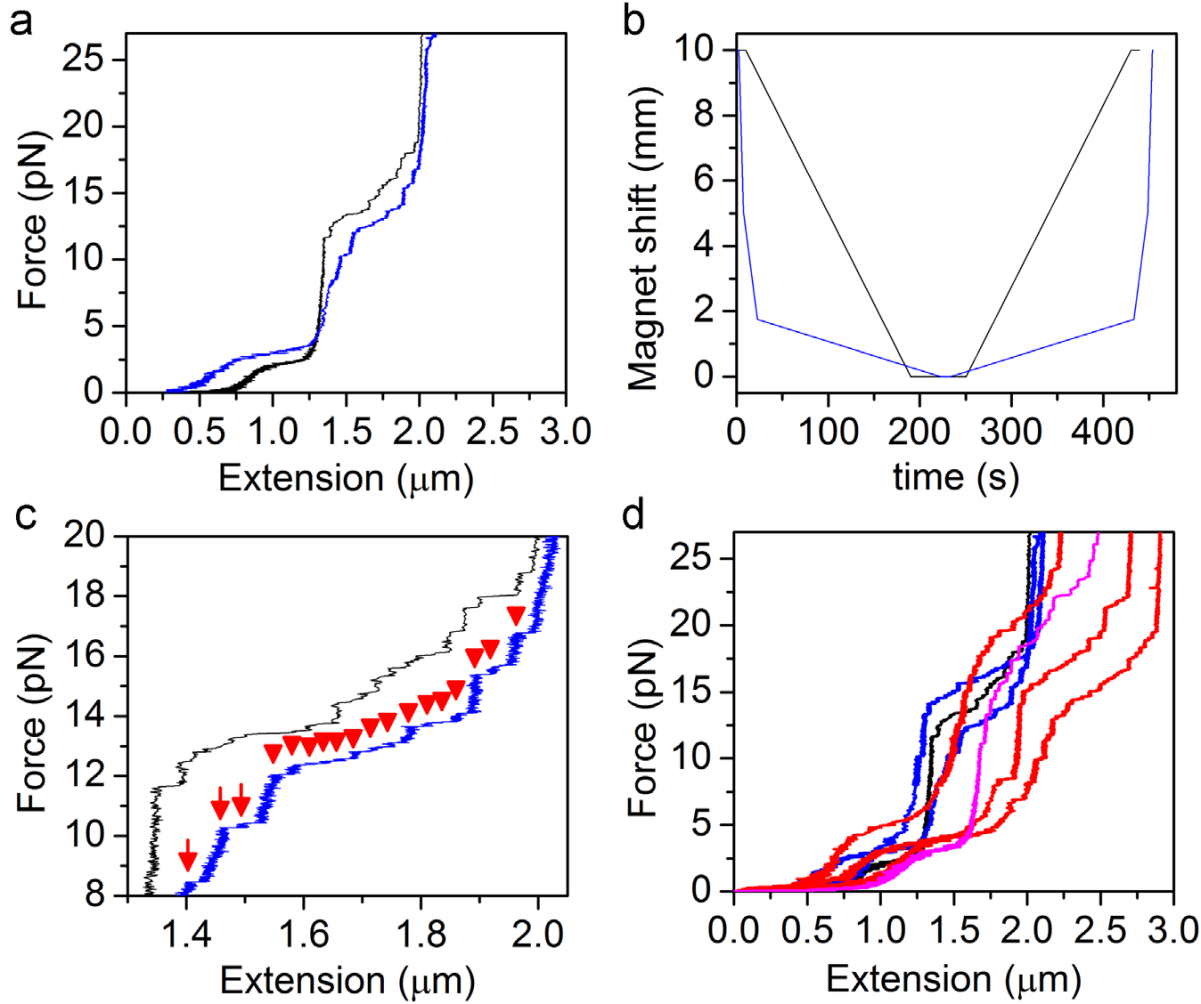
Force–Extension traces at the force regime from 0 pN to 27 pN. (a) FE traces of SS Chromatin fibers stretched at CMS (black line) and at PMS (blue line). (b) Trajectories of CMS (black line) and PMS (blue line). (c) Close-up of the stepwise rupture events in the high force regime between 8 and 20 pN. The red arrows point to individual rupture events. The FE trace measured at a constant magnet shift rate (black line) shows larger steps compared to the trace measured at a programmed magnet shift rate (blue line). (d) Force–Extension traces of SH chromatin fibers measured at CMS (the black line) or at PMS (blue lines). The magenta line shows the Force–Extension traces of LS chromatin fibers measured at CMS. Three Force-Extension traces of LH chromatin fibers measured at PMS are represented as red lines.

**Fig. 2:**
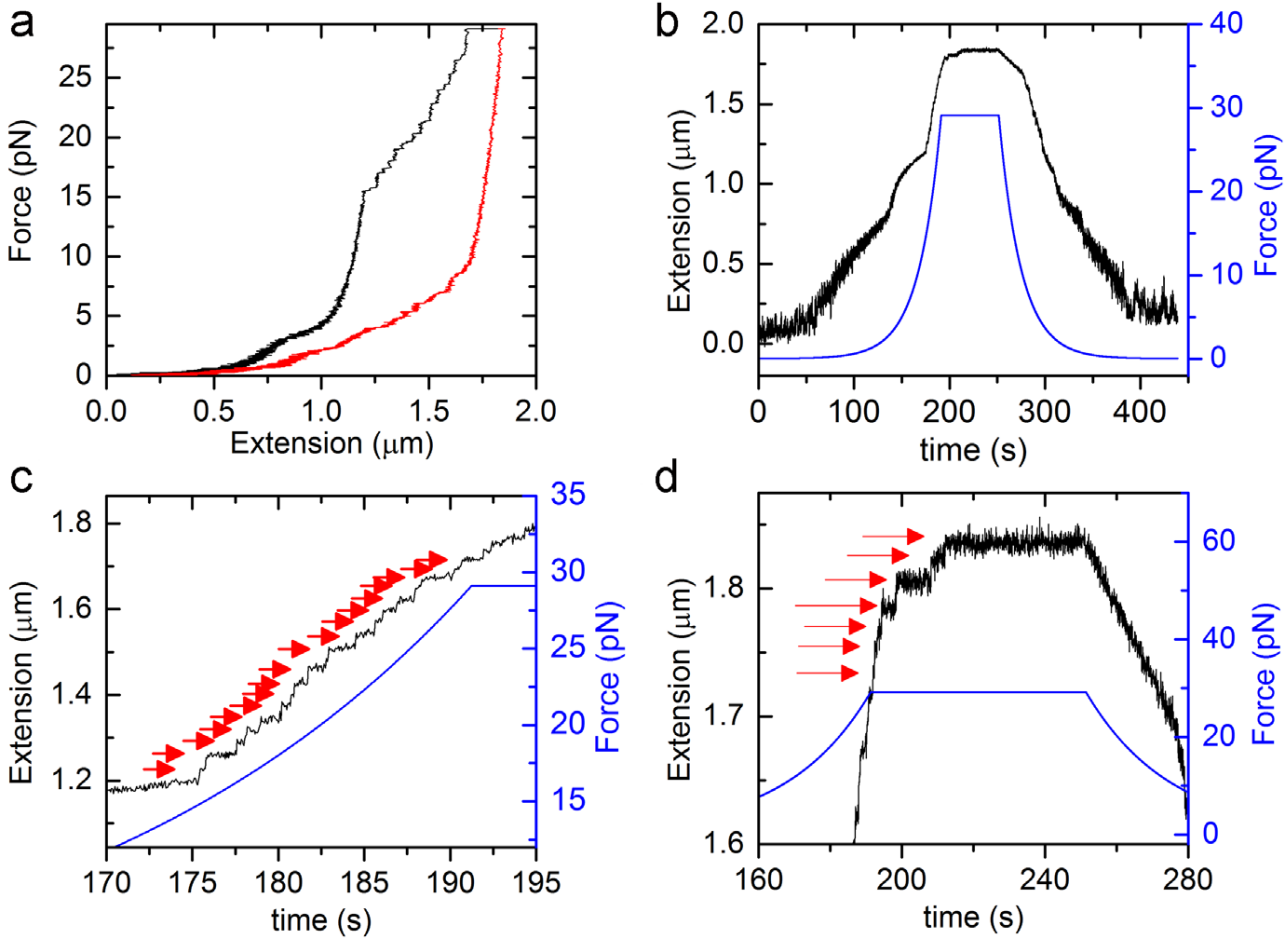
One single chromatin fiber in one pulling cycle. (a) Force-Extension traces of a SH chromatin fiber in the presence of an increasing force (black line) and a decreasing force (red line). (b) The measured length of the SH chromatin fiber is shown as the black line. The blue line shows the corresponding exerted force. (c) Close-up of the stepwise rupture events (black line) indicated with red arrows in the force regime between 15 pN and 29 pN. (d) Close-up of the stepwise rupture events (black line) pointed with red arrows at the constant force of 29 pN.

Chromatin fibers were reconstituted with purified chicken histone octamers and DNA fragments containing 601 sequences through salt dialysis described previously [11]. One extremity of a single chromatin fiber was attached to the surface of the cover slip and the other extremity is tethered to a paramagnetic bead with the diameter of 2.8 µm [5]. Short DNA spacers of 251 bp (SS) and long DNA spacers of 2360 bp (LS) were applied to prevent chromatin fiber from sticking to the glass surface [12]. The exerted force is corresponding to the magnet position and calculated with the method described previously [13]. In order to capture the details of single nucleosome unwrapping events, we changed the constant magnet shift (CMS) to the programmed magnet shift (PMS). The rate of CMS is 10 mm/180 s and PMS contains three rates of 5 mm/5 s, 3.25 mm/30 s, and 1.75 mm/200 s. Figure 1 shows the details of the Force-Extension traces of the SS and LS chromatin fibers measured at CMS or PMS mode. These fibers showed no rapture events at the highest exerted force. However, the stepwise rupture events continued at the highest force in the chromatin fiber shown in Fig. 2. The process of chromatin refolding in the presence of decreasing force was recorded in Fig. 2.
